# Does structured counselling influence combined hormonal contraceptive choice?

**DOI:** 10.3109/13625187.2011.625882

**Published:** 2011-11-08

**Authors:** Mireille Merckx, Gilbert G Donders, Pascale Grandjean, Tine Van de Sande, Steven Weyers

**Affiliations:** * Universitair Medisch Centrum St Pieter, VUB/ULB, Brussels; † Heilig Hart Ziekenhuis, Tienen; ‡ Universitair Ziekenhuis Leuven, Leuven; § Centre Hospitalier Régional de Mons, Mons; # Medical Department MSD Belgium, Ghent, Belgium; ^ Universitair Ziekenhuis Gent, Ghent, Belgium

**Keywords:** Combined hormonal contraception, Combined oral contraceptives, Transdermal patch, Vaginal contraceptive ring, Counselling

## Abstract

**Objective:**

To assess the effect of structured counselling on women's contraceptive decisions and to evaluate gynaecologists’ perceptions of comprehensive contraceptive counselling.

**Methods:**

Belgian women (18–40 years old) who were considering using a combined hormonal contraceptive (CHC) were counselled by their gynaecologists about available CHCs (combined oral contraceptive [COC], transdermal patch, vaginal ring), using a comprehensive leaflet. Patients and gynaecologists completed questionnaires that gathered information on the woman's pre- and post-counselling contraceptive choice, her perceptions, and the reasons behind her post-counselling decision.

**Results:**

The gynaecologists (*N* = 121) enrolled 1801 eligible women. Nearly all women (94%) were able to choose a method after counselling (53%, 5%, and 27% chose the COC, the patch, and the ring, respectively). Counselling made many women (39%) select a different method: patch use increased from 3% to 5% (*p* < 0.0001); ring use tripled (from 9% to 27%, *p* < 0.0001). Women who were undecided before counselling most often opted for the method their gynaecologist recommended, irrespective of counselling.

**Conclusion:**

Counselling allows most women to select a contraceptive method; a sizeable proportion of them decide on a method different from the one they initially had in mind. Gynaecologists’ preferences influenced the contraceptive choices of women who were initially undecided regarding the method to use.

## INTRODUCTION

In Western countries, there is a wide choice of contraceptive options. Yet, abortion rates remain unacceptably high and are even rising. In Belgium, where abortion is legally permitted, the reported abortion rate of about one in 100 women per year is among the lowest in the world^1^-^3^, and the lifetime risk of having an induced abortion is about one in six^2^. Half of the women seeking an abortion in Belgium were not using reliable contraception, 15% relied only on condoms, and 26% were taking a combined oral contraceptive (COC). These figures are consistent with those from the United States (US), where up to 20% of all unwanted pregnancies are due to the incorrect or inconsistent use of oral contraceptives^4^. Trussell recently showed that in the US the unintended pregnancy rate during the first year of 'typical use’ is 9% for all types of combined hormonal contraceptives (CHCs)^5^. While irregular use was thought to be most common among adolescents and young women, recent research indicates that non-use and poor compliance are common in *all* age groups^6^. Lack of compliance is related to deficient knowledge^7^ and poor motivation. Another, possibly underestimated, reason is women's dissatisfaction with their chosen contraceptive method.

During the last decade, two alternatives to COCs have expanded women's options: the CHCs concerned are the transdermal patch and the vaginal ring. The patch is replaced once per week; the ring once per month. A recent survey by Lete *et al*. showed a substantial improvement in compliance in users of these new non-daily methods: 68% and 78% of patch- and ring users, respectively, reported consistent use compared with only 29% of COC users^8^.

However, widening the range of CHC options with methods not requiring a daily intervention may not suffice to increase compliance. Glasier *et al*. showed that neither the wide availability of contraceptive methods nor the free provision of emergency contraception changed women's behaviour or reduced the need for abortion^9^. While effective counselling is crucial to maximise contraceptive compliance^10^, ^11^, the wide range of products available today makes counselling more difficult for the clinician^12^, ^13^. Easy-to-use counselling tools such as information leaflets can assist healthcare professionals and women during counselling sessions^6^.

The Contraceptive Health Research Of Informed Choice Experience (CHOICE) study was initiated in 11 countries to encourage healthcare professionals (HCPs) to study and improve counselling of women contemplating the use of a CHC. It assesses the influence a standardised counselling guide may have on women's contraceptive decisions and evaluates how the method finally chosen by women differs from that they originally thought they would employ. In Belgium, the CHOICE study included an additional questionnaire that was offered to HCPs to assess whether they preferred structured contraceptive counselling and/or the use of the specially designed leaflet over their usual contraceptive counselling approach.

## MATERIALS AND METHODS

The cross-sectional, multinational CHOICE study involved 11 countries with very different contraceptive service provision and practices: eight European countries (Austria, Belgium, the Czech Republic,The Netherlands, Poland, Slovakia, Sweden, and Switzerland), Israel, the St. Petersburg and Moscow regions of the Russian Federation, and Ukraine. The target for Belgium was to include 1850 women between 18 and 40 years old. In Belgium, only gynaecologists were asked to participate. Gynaecologists (*N* = 121) were expected each to recruit ten or more women whom they would see during hospital consultations or in their individual practices. Gynaecologists kept a log of *all* women consulting for contraception during the study period regardless of whether they were enrolled in the CHOICE study or not. Women who considered starting a CHC method or switching from one CHC method to another were invited to participate. Women who *a priori* excluded one or more of the three methods (COC, patch or ring, possibly because they were not satisfied with their current method and wanted to switch to another CHC) were not eligible to participate. A counselling leaflet presented information about the different types of CHCs, including their mode of action, mode of administration, benefits and side effects. The counselling leaflet, which was derived from a leaflet used in the TEAM-06-study by Lete *et al.^14^,* was prepared in cooperation with the European Society of Contraception and Reproductive Health (ESC) and was offered to the clinician for use during counselling. If, during the consultation (i.e., when the woman was being invited to participate in the CHOICE study) the gynaecologist believed that another (non-CHC) method was more appropriate, the counselling leaflet was not used but the study questionnaires were still completed. The study was approved by the central ethical committee of Ghent University Hospital and subsequently by all other required local ethics committees. All participating women gave written informed consent prior to enrolement. A local Belgian steering committee (made up of four of the authors of this manuscript: MM, GD, PG, SW) supervised the study from start to finish.

Before the counselling session, the gynaecologist asked the woman if she already had a preference for any CHC. The gynaecologist then counselled the woman about all three CHC options (and/or other methods if deemed suitable). Use of the counselling leaflet was optional but recommended. The content of the counselling guide was well known to all the gynaecologists, and if they — for one or more reasons — decided not to use the counselling leaflet during the contraceptive discussion, they were nonetheless supposed to provide their patients with the same information and to counsel them as extensively as possible on each of the three methods. The gynaecologist checked whether contraindications existed for any of the CHC methods and documented on the questionnaire whether the counselling leaflet had been used. The patient provided demographic information and rated various characteristics of the CHC methods described to her by her gynaecologist. She also indicated which method she ultimately chose and the reasons for her choice. The questionnaire included 18 questions and took about ten minutes to complete.

### Statistics and sample size

A primary statistical objective of the study was to determine with sufficient precision the selection rates of the pill, patch, ring or other method after counselling or whether the woman was still undecided. A precision of 2% (the half-width of the simultaneous two-sided 95% confidence interval [CI] for choosing each the weekly patch or monthly ring) was selected; it was also assumed that 10% of women in each country would select the patch and 10% would select the ring after counselling. This resulted in 1500 required participants per country.

A secondary statistical objective of the CHOICE study was to demonstrate that the selection of a method other than the pill (e.g., patch or ring) undergoes a statistically significant increase after contraceptive counselling compared with the woman's pre-counselling contraceptive choice. For the patch and the ring, we aimed at detecting differences of at least 3% between post-counselling and pre-counselling contraceptive choices. It was assumed that 5% of the women who chose the patch and 5% of those who chose the ring prior to counselling would change their mind and select another method after counselling. Statistical power analysis on this secondary objective led to the determination that 1070 women needed to participate in each country to yield a power of 90% to detect an increase of at least 3% in either the selection of the patch or ring, and maintain a false-positive (or type I) error of 5%. Since two comparisons (one for the patch and one for the ring) were required, a one-sided statistical significance level of 1.25% was used.

After accounting for these considerations, we determined that we would need to recruit at least 1500 participants in each country to meet the statistical objectives of the CHOICE study. The sample size needed to be adjusted upwards by about 20% to compensate for non-evaluable questionnaires and erroneous study entry, resulting in a target sample size of 1850 women.

For the post-counselling selection of contraceptive methods, simultaneous 95% CIs were calculated based on the 5-cell multinomial probability distribution. The difference in proportions between the chosen and the intended methods is presented with the two-sided 97.5% CI for the patch and the ring. The statistical significance of these differences was assessed using McNemar's test for differences in proportions. All other analyses are exploratory and a two-sided significance level of 5% was used.

The questionnaires included questions about women's perceptions regarding the efficacy, safety and use aspects of the three CHC methods after counselling. To assess the association between these perceptions and whether or not women decided to use the method concerned, the probability of choosing a method was modelled against agreement or disagreement with the perception statements (with the categories ‘no opinion’ and ‘do not know’ as a combined reference category). The participant's age was included in the models as a covariate.

## RESULTS

The characteristics of the participating gynaecologists are summarised in Table 1. Most of the gynaecologists were women (56%) and one in three was more than 49 years old. HCPs were most likely to recommend COCs to women who were consulting for contraception (90%), followed by the levonorgestrel releasing-intrauterine system (LNG-IUS, 5%).

**Table 1:** Gynaecologists’ characteristics.

	*n*	*%*	*Mean*	*SD*	*Median*	*Range*
*Total number of gynaecologists who enrolled subjects*	121					
*Gender*	119*					
Female	67	56				
Male	52	44				
*Age (years)*	119*					
20-29	0					
30-39	41	35				
40-49	38	32				
50-59	25	21				
60 and above	15	13				
*Consultations for contraception per week on average*	118#		35.1	20.7	30	5-150
*Requests for CHC method per week on average*	118#		24.3	13.7	20	1-68
*Most frequently recommended contraceptive method*	115$					
Combined oral contraceptive	103	90				
Vaginal ring	4	4				
Levonorgestrel releasing-intrauterine system	6	5				
Copper-intrauterine device	1	1				
Progestogen-only-pill	1	1				
Condoms	0					
Transdermal patch	0					
Contraceptive implant	0					
Natural family planning	0					
Injectable	0					
Sterilisation	0					

* Missing data *n* = 2.

# Missing data *n* = 3.

$ Missing data *n* = 6; Condoms, patch, contraceptive implant, natural family planning, injectable and sterilisation were never mentioned by the participating gynaecologists as the contraceptive method they most frequently recommended.

Of all the collected questionnaires (*N* = 1843), 42 (2%) were excluded from analysis because of violation of the country-specific age criterion (≥18 years and ≤40 years old). This resulted in a study population of 1801 eligible women.

The log of women, in which gynaecologists were to register *all* women consulting for contraception during the study period, comprised 5906 women. Of the women who figured in this log, 1437 (24%) were included in the study because they requested a CHC *and* fulfilled the CHOICE study inclusion criteria. This shows that the log was *not* filled out systematically for *all* women presenting for contraceptive advice: indeed, only 1437 of the 1801 women included in the study could be traced back to the log.

Table 2 shows the reasons for contraceptive consultation of all consulting women according to the log. Women enrolled in the study were more likely to have problems with or questions about their current contraceptive method; they were also more likely to consider starting or switching to a new method than women who were not enrolled.

**Table 2:** Log of women consulting for contraception.

	*Enrolled in CHOICE?*			
	
Reason for contraceptive consult*	*Yes 1437 (24%)*	*No 4367 (74%)*	*Missing answer 102 (2%)*	*n 5906*
Repeat prescription	497 (35%)	1768 (41%)	35 (34%)	2300
Periodic check	435 (30%)	2360 (54%)	49 (48%)	2844
Problem with current contraceptive	319 (22%)	351 (8%)	9 (9%)	679
Questions about current contraceptive	272 (19%)	304 (7%)	8 (8%)	584
Initiation of contraception or switch to other method	410 (29%)	374 (9%)	12 (12%)	796
Emergency contraception	7(1%)	36 (1%)	0	43
Other	42 (3%)	459 (11%)	8 (8%)	509

* Multiple answers possible.

Characteristics of participants included in the analysis are summarised in Table 3. Two out of three participants had a high educational level and over 70% were employed. About one in four (27%) women did not want to have more children. Unintended pregnancies were reported by 9% of the women. The most commonly last-used main contraceptive method was the COC (67%).

**Table 3:** Patients’ characteristics.

	*n*	*%*	*Mean*	*SD*
Age (years)*	*1800*		278	6.3
≤20	274	15		
21-25	442	25		
26-30	472	26		
31-35	341	19		
36-40	271	15		
*Highest educational level*	*1796*			
Primary school	34	2		
Secondary school	581	32		
Advanced, non university	826	46		
University	355	20		
*Employment status*	1769			
Unemployed	518	29		
Part-time	263	15		
Fulltime	988	56		
*Future desire for children*	1792			
No	479	27		
Yes	1027	57		
Do not know yet	286	16		
*Unplanned pregnancies*	1792			
No	1638	91		
Yes	154	9		
1	117	84		
2	19	14		
>2	3	2		
Missing data	15			
*Steady relationship*	*1798*			
No	257	14		
Yes	1541	86		
*Last main contraceptive*	*1788*			
*method*				
Combined oral	1206	67		
contraceptive				
Vaginal ring	128	7		
Condoms	94	5		
Progestogen-only-pill	91	5		
LNG releasing-intrauterine	88	5		
system				
Never used contraception	72	4		
Transdermal patch	39	2		
Copper-intrauterine device	35	2		
Contraceptive implant	22	1		
Natural family planning	11	1		
Injectable	2	0		

* For one patient the age was not mentioned, nevertheless she was included in the full analysis since missing age was not an exclusion criterion.

The leaflet was used during 80% of the counselling sessions. Most women rated the leaflet as ‘somewhat’ or ‘very' useful (94%), 'complete’ (91%) and 'fair/balanced’ (94%). Nearly all participants (*n* = 1790 [out of 1801]; 99%) answered the questions about their pre- and post-counselling contraceptive preferences. Women's intended and chosen methods are shown in Table 4. A total of 703 women (39%) with a preconceived idea regarding which method they intended to use before counselling changed their contraceptive preference after counselling. Women who initially preferred the COC or the vaginal ring were the least likely to change their choice (69% and 86% of those preferring pill or ring, respectively, did not change their contraceptive method after counselling). Although only 9% of women contemplated using the ring prior to counselling, 27% selected that contraceptive after counselling (difference in proportions = 18%, 97.5% CI 16-20%, p < 0.0001 McNe-mar's test). The patch was chosen twice as often after counselling (5.2% vs. 2.6% before counselling; difference = 2.7%, 97.5% CI 1.4-3.9%, *p* < 0.0001 McNe-mar's test). The pill was chosen 14% less often after counselling (53% vs. 67% before counselling; difference =-14%, 95% CI -17 to -12%, *p* < 0.0001), but still remained the most frequently chosen method. Of women switching from the pill to another method (*n* = 366), 14% chose the patch, and 57% the ring. There were no differences in final choices nor in changes between initial preference and final choice between women counselled *with* or *without* use of the leaflet (data not presented).

**Table 4:** Cross tabulation of method the woman intended to use before counselling and method chosen after counselling

*n = 1799*	*n (%)*	*Pill chosen*	*Patch chosen*	*Ring chosen*	*Other method chose*	*Not decided yet*	*Missing data*
Patient had no initial preference	199 (11%)	59 (30%)	18 (9%)	**63 (32%)**	32 (16%)	25 (13%)	2
Patient intended to use pill	1202 (67%)	**830 (69%)**	52 (4%)	209 (18%)	42 (4%)	63 (5%)	6
Patient intended to use patch	47 (3%)	6 (13%)	**20 (44%)**	16 (35%)	2 (4%)	2 (4%)	1
Patient intended to use ring	164 (9%)	8 (5%)	0	**141 (86%)**	14 (9%)	1 (1%)	0
Patient intended to use other method	187 (10%)	38 (20%)	4 (2%)	53 (28%)	**71 (38%)**	21 (11%)	0
Method chosen	1799	941 (53%)	94 (5%)	482 (27%)	161 (9%)	112 (6%)	9

Figures highlighted in grey: Non-changers							
**Figures in bold:** Method most frequently chosen in relation to the initial preference							

Cross tabulation of method which the gynaecologist thought was best for the woman without initial preference and method chosen after counselling							

*n = 156*	*n (%)*	*Pill chosen*	*Patch chosen*	*Ring chosen*	*Other method chosen*		

Had no initial preference	83 (53%)	35 (42%)	7 (8%)	**26 (31%)**	15 (18%)		
Thought pill was best	12 (8%)	**10 (83%)**	0	1 (8%)	1 (8%)		
Thought patch was best	6 (4%)	1 (17%)	**5 (83%)**	0	0		
Thought ring was best	40 (26%)	4 (10%)	3 (8%)	**29 (73%)**	4 (10%)		
Thought other method was best	15 (10%)	2 (13%)	1 (7%)	1 (7%)	**11 (73%)**		

Figures highlighted in grey: Same method chosen as the one the gynaecologist intended to prescribe

**Figures in bold:** Method most frequently chosen in relation to the method the gynaecologist thought was best for the patient

Nearly all (*n* = 172; 87%) of the 197 participants who were undecided before counselling had a contraceptive preference after counselling. Of these women, 34% chose the pill, 10% the patch, and 37% the ring. To evaluate if gynaecologists might have had an influence on the final choice of women who were undecided prior to counselling, we investigated the relationship between the method chosen by the woman and that which the gynaecologist thought was best for her. Information was available for 156 of 199 women (78%) who were undecided. The gynaecologist had no preconceived preference in 53% of these cases; for the remaining 47% of undecided women, the pill, patch and ring were the gynaecologists’ recommended method in 8%, 4%, and 26% of the cases, respectively. When the gynaecologist had a preference for a particular method but the patient did not, the gynaecologist's preferred method was adopted by the participant in 83% of the cases for the pill and the patch, 73% for the ring, and 73% for other methods. If the gynaecologist had no preconceived preference, participants most often chose the pill (42%) or the ring (31%).

**Figure 1:**
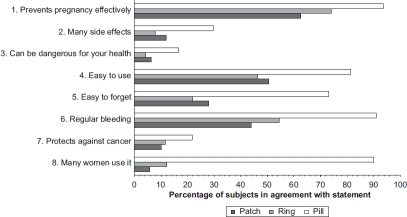
Patients’ opinions on contraceptive methods.

Women's perceptions about the three CHC methods are shown in Figure 1. All three methods were seen as very effective. However, women were much less knowledgeable about the patch and the ring than about COCs, even after counselling. In the opinion of the participants, the patch and ring are less easily forgotten than the pill.

Table 5 shows the age-adjusted relation between women's perceptions of a certain method and the likelihood of adopting it. There was a non-significant positive trend between the woman's age and the probability of choosing the ring. On the other hand, there was a 5—6% statistically significant (p < 0.0001) decline in the probability of choosing the pill per five-year increase of age (data not shown). Agreement with the statement ‘prevents pregnancy effectively’ increased the probability that women chose that method compared to women who had ‘no opinion’ or ‘did not know’ of the method. Lack of confidence in the contraceptive efficacy of the ring decreased the probability that a woman would choose it by 11% (*p* = 0.015). Paradoxically, even women who had no confidence in the efficacy of COCs still were 18% more likely to select the pill compared with women who had ‘no opinion’ or ‘did not know’ (*p* = 0.044).

**Table 5:** Summary results of the binomial regression models for prediction of the choice of contraceptive method#

	(*Strongly) agree*		(*Strongly) disagree*	
		
	*Estimate % (95% CI)*	*p-value*	*Estimate % (95% CI)*	*p-value*
Prevents pregnancy effectively	17 (12.8-21.3)	< 0.0001	−11 (−19.1-−2.0)	0.015
Has many side effects	−9 (−15.0-−3.0)	0.0034	21 (16.4-26.0)	< 0.0001
Can be dangerous for health	−1 (−10.2-9.3)	0.93	12 (77-16.2)	< 0.0001
Is easy to use	27 (22.1-30.9)	< 0.0001	−13 (−16.6-−9.2)	< 0.0001
Is easy to forget	−4 (−8.5-1.3)	0.15	17 (12.4-22.1)	< 0.0001
Gives regular bleeding Protects again cancer	19 (15.3-23.4)	< 0.0001	14 1 (−3.6-31.9) (−4.3-71)	0.12
Protects again cancer	−0 (−6.7-6.5)	0.98	1(−4.3-7.1)	0.63
Is used by many	15 (77-21.8)	< 0.001	−5 (−10.1-−0.6)	0.026

*Patch*	Estimate % (95% CI)	p-value	Estimate % (95% CI)	p-value

Prevents pregnancy effectively	6 (3.8-76)	< 0.0001	2 (−3.0-6.4)	0.48
Has many side effects Can be dangerous for health	1 (−1.9-4.3)	0.45	8 (4.2–11.8)	< 0.0001
Can be dangerous for health	3 (−1.9-8.3)	0.22	3 (0.2–4.8)	0.034
Is easy to use	*			
Is easy to forget	0 (−2.0-2.1)	0.95	7 (4.3-10.4)	< 0.0001
Gives regular bleeding	4 (1.3-5.7)	0.0018	8 (−4.2-19.5)	0.21
Protects again cancer	−2 (−4.9-1.1)	0.21	1 (−2.2-3.9)	0.58
Is used by many	15 (71-22.7)	< 0.001	−1 (−3.1-1.2)	0.37

*Pill*	Estimate % (95% CI)	p-value	Estimate % (95% CI)	p-value

Prevents pregnancy effectively	26 (14.9-372)	< 0.0001	18 (0.5-35.0)	0.044
Has many side effects	−17 (−23.1-− 11.0)	< 0.0001	14 (8.2–19.4)	< 0.0001
Can be dangerous for health	−8 (−15.3−-1.5)	0.017	12 (71-175)	< 0.0001
Is easy to use	31 (24.6-38.0)	< 0.0001	−10 (−19.0-−1.7)	0.019
Is easy to forget	−25 (− 31.6-−18.6)	< 0.0001	4 (−3.4–12.3)	0.27
Gives regular bleeding	20 (10.1–30.7)	0.0001	−1 (−15.4–12.8)	0.86
Protects again cancer	9 (3.6-15.0)	0.0015	−0 (−6.0–5.4)	0.92
Is used by many	2 (−6.3-9.7)	0.67	−20 (−45.4-5.1)	0.12

The estimates for ‘(Strongly) agree’ and ‘(Strongly) disagree’ reflect the difference in probability to select the method with respect to the category ‘No opinion'/'do not know'.

* Could not be calculated due to a zero in the category ‘(strongly) disagreed'.

# Results are corrected for age (age of the subject was included in the models as a covariate).

Dark grey: statistically significant negative impact.

Light grey: statistically significant positive impact.

Women who disagreed with the statements ‘has many side effects’ and/or'can be dangerous for health’ had a higher probability of choosing that method than those with ‘no opinion’ or ‘did not know'. In addition, women who presumed that a method was associated with a regular bleeding pattern were significantly more likely to choose that method. Women who were aware of the reduced likelihood of forgetting a certain method had an increased tendency of choosing the patch or the ring (increased probability of 7% and 17%, respectively,*p* < 0.0001), while those who believed that the pill is easy to forget were 25% less likely to choose this method (*p* < 0.0001). An opinion that a method ‘is used by many women’ did not significantly influence women's selection of the pill, but it augmented the probability of selecting the patch or the ring by 15% for either of these methods (*p* < 0.001).

More than 85% of the gynaecologists found that the counselling leaflet was complete (Table 6). Nearly 90% of them found that it was not useful during their own counselling sessions, although 54% agreed that the leaflet would be useful for the woman during the counselling process.

**Table 6:** Perception of gynaecologists about the structured counseling (with counselling leaflet) as used in CHOICE

	*More*	*Equal*	*Less*
Did the counselling take more time/	115 (46%)	131 (53%)	2 (1 %)
Did you inform her about more contraceptive methods/	93 (38%)	152 (62%)	1 (< 1 %)
Did you give the woman more freedom to choose/*	16 (7%)	224 (91%)	3 (1 %)
Did you pay more attention to her medical history/	16 (7%)	225 (92%)	2 (1 %)
Did you pay more attention to her medical conditions/	12 (5%)	228 (94%)	2 (1 %)
Did you pay more attention to possible contra-indications/	11 (5%)	229 (94%)	3 (1 %)
Did you pay more attention to special contraceptive needs/	40 (17%)	198 (83%)	1 (< 1 %)
	*Yes*	*No*	
Did you have a more open discussion/	37 (15%)	203 (85%)	
Did the counselling process uncover factors that made you change your precounselling opinion/	210 (14%)	34 (86%)	
Was the counselling more useful for the women/	132 (54%)	111 (46%)	
Was the counselling more useful for the gynaecologist/	27 (11%)	216 (89%)	
Was the counselling more time-consuming/	133 (54%)	112 (46%)	
Was the counselling leaflet complete/**	207 (86%)	29 (12%)	

* 1% of answers missing.

** 2% of answers missing.

## DISCUSSION

The cross-sectional, multinational CHOICE study involved eight European countries (Austria, Belgium, the Czech Republic, The Netherlands, Poland, Slovakia, Sweden, and Switzerland), Israel, the St. Petersburg and Moscow regions of the Russian Federation, and Ukraine. Contraceptive patterns and prevalences vary widely among these countries and are very much influenced by the providers of contraception (gynaecologists, general practitioners [GPs] or other HCPs), prescribing guidelines, reimbursement arrangements, prevailing opinions about the various contraceptive methods and - in Central and Eastern Europe - political and social changes. In this spectrum, Belgium represented a country with wide availability of hormonal contraceptives, affordable consultations (most of the consultation costs are covered by the health insurance system), where gynaecologists are the main providers of contraceptive counselling and abortion rates are very low.

This study mainly investigated the influence of a structured counselling session with or without a Counselling Leaflet on the choice of women considering the use of a CHC. The information in the leaflet was limited to CHC methods only, thus excluding information on alternative methods such as progestogen-only methods and intrauterine systems (IUSs). It was left to the discretion of the gynaecologist to give information regarding other contraceptive options. Women more than 40 years old were excluded from participation as they were thought to benefit from a broader contraceptive range (IUS, sterilisation), even if they considered using a CHC. The leaflet employed was conceived in collaboration with, and endorsed by, the ESC and an international steering committee of experts in contraception.

According to the log, about 40% of women in the study had a problem with or questions about their current contraceptive method; 29% also considered starting or switching to a new method. The subset of women enrolled in the study contained a selection of new starters or re-starters of a CHC method, and thus differed from the average female population consulting for contraceptive reasons, as demonstrated by the log.

Despite the availability of newer CHCs such as the patch and ring, the CHC method most frequently prescribed by the participating gynaecologists was still the COC. The pill is indeed the method best known to physicians and women, which makes counselling easy and swift. In addition, cost factors may favour the pill since COCs are available in a wide range of prices, and many of these are cheaper and better reimbursed than other CHCs. Moreover, in Belgium, some COCs are available free of charge for women aged less than 21. All other women need to pay. Since older COCs are partially reimbursed, the difference in price between the pill and other CHCs (ring and patch) can be considerable.

This study confirmed that the pill was the most commonly used contraceptive method in Belgium, even in a population of women who were not entirely satisfied with their current or previous family planning (FP) method (about two in three women included in the CHOICE study had been using a COC as their last FP method). Prior to counselling, a similar proportion of women (67%) wanted to start a COC. However, counselling had an undisputable influence on women's ultimate decisions: 39% of the women with a preference prior to counselling switched to a new method after counselling. Ultimately, 53%, 5%, and 27% of women opted for the pill, the patch, and the ring, respectively.

Only one in four women did not want to have more children. Thus, a vast majority of the study participants were looking for an easily reversible contraceptive method. Only 9% of women reported having had an unintended pregnancy, while 6% had undergone an induced abortion. The latter figure is probably an underestimation since 6% of the women did not answer this question. The specific age inclusion criteria (< 40 years old) may have influenced the abortion rate: women older than 40 years have relatively high abortion rates^15^-^16^. In the United States, the unintended pregnancy rate and abortion rate above the age of 40 years are 5/1000 and 3.2/1000 women per year, respectively^15^. In Belgium, 23% of pregnancies in women who are over 40 years old end in an abortion^16^. Equally important is the high average educational level of the women enrolled in the Belgian arm of the CHOICE study: only 2% of them had not completed secondary school. In the United States, unintended pregnancy rates are substantially higher in women who did not complete high school, and while these rates in general declined between 1994 and 2001, they increased in women with a lower educational level^17^. Finally, the large number of participants who reported being in a stable relationship (85%) could have contributed to the low abortion rate observed in this study.

The counselling process, with or without the use of the counselling leaflet, was considered useful by nearly all women. No differences were observed in final choice between women counselled with and without the leaflet. This is probably due to the fact that gynaecologists were instructed to give complete information about all three types of CHC, as if they would have used the counselling guide. The existence of a structured leaflet shown to the women did not seem to have a great influence. Eventually, almost all of the women (94%) were able to select a method after counselling. Of all women, 40% did not stick to the contraceptive method they originally had in mind; the remaining 60% were not influenced by the counselling process. While two in three women consulted their gynaecologist with the idea of starting a (new) COC, just over half (53%) of them actually did so after counselling. The ring was chosen three times more frequently than originally intended. The changes between women's preconceived preference and final choice, which were statistically significant, indicated that the counselling sessions greatly influenced women's selection of contraceptives. Among those without a clear preference before counselling, the ring was even more frequently chosen than the pill. In women who switched from the pill during counselling, the ring was by far the most often method adopted after counselling. Although not included in the counselling guide, 6% of women chose the LNG-IUS after counselling.

In general, participating women had a high degree of confidence in the contraceptive effectiveness of CHCs, especially that of COCs. Surprisingly, both women who (strongly) disagreed and those who (strongly) agreed that the pill is highly effective had a higher probability of choosing this method. Apparently, even women who believe that the pill can be easily forgotten still opt for this method. This may be due to the fact that the Belgian women in the CHOICE study - as the latter demonstrated - were highly knowledgeable about COCs, including their advantages and disadvantages; their knowledge of the pill was certainly greater than that of other CHCs.

The selection of the patch and the ring was significantly associated with the belief that the interventions required with these methods will be less easily forgotten. This is consistent with previous findings that patch- and ring-users may demonstrate greater compliance compared with pill-users^8^.

Although contraception is prescribed both by gynaecologists and GPs in Belgium, only gynaecologists were asked to participate in the study. In Belgium, gynaecologists during their consultations see more women seeking advice regarding CHCs (on average, 24 women per week; Table 1) than GPs, which facilitated recruitment. Prior to the study, we believed that women's contraceptive choices are influenced predominantly by the media and peers. One of the remarkable results of the study was the impact of the gynaecologist on the contraceptive decisions of women who were undecided prior to counselling. When, in those circumstances, the gynaecologist had a preference, it was usually adopted by the woman. This suggests that the relationship between women and their gynaecologists in Belgium is characterised by much confidence and trust. This may be of particular interest, as participant compliance has, to a great extent, been linked to the patient's satisfaction with the clinician. In all circumstances, it is of considerable importance that the clinician fully adapts the contraceptive counselling to the participant's individual needs, lifestyle, concerns and expectations^18^-^19^. This entails that a dialogue should take place, which may help to ensure the participant's understanding and correct interpretation of all information provided during the consultation. On top of this, easy-to-understand literature, specifically conceived for patients (including leaflets) and containing all relevant information, may also be extremely useful according to some authors^20^, ^21^.

The counselling guide used in the current study was considered complete by a large majority of gynaecologists. However, the Belgian gynaecologists also felt they did not really need a structured counselling guide that could assist them with the counselling process. Paradoxically, gynaecologists agreed that such a guide helps women who are counselled. Half of the gynaecologists found standardised counselling using the leaflet to be more time-consuming than the method they normally resorted to during counselling. Nevertheless, their overall responses indicated that the majority of gynaecologists found that the structured and broad counselling process that was used in the CHOICE study was more useful to women (which should be their ultimate goal). An open dialogue between clinicians and women seeking contraception, whether based on a structured leaflet or not, will lead to the selection of a contraceptive method that is better suited to a woman's needs. In the end, this may ultimately lead to increased satisfaction and improved compliance among CHC users.

One of the limitations of the study is its cross-sectional design. Since follow-up visits were not included in the study, we could not evaluate compliance for the chosen method or continuation rates. Another limitation was that the information in the leaflet was limited to CHCs. The study thus focused on women who were considering CHC methods and who were 18-40 years old. We excluded women who were considering (or for whom the gynaecologists considered) alternative methods such as progestogen-only methods or intra-uterine systems (IUSs), as well as younger and older women. Inclusion of these other populations would have complicated the study and made statistical analysis more difficult. Moreover, far more women would have been necessary to meet the primary statistical objective. The counselling leaflet included reference to progestogen-only methods, and when the gynaecologist considered during a consultation of a woman with initial interest in a CHC method who was included in the study, that another method was more suitable, they counselled about alternative methods. This methodology was endorsed by the ESC.

We did not observe how the gynaecologists actually counselled the women but relied on our instructions to the participating gynaecologists on how to counsel their patients. The survey among gynaecologists indicated that many used the counselling method as instructed. We noted that the gynaecologists had a profound influence on the contraceptive choice of women who were initially undecided: 80% of these adopted the gynaecologist's preferred method. Finally, a last limitation could be the fact that only gynaecologists participated in this study, while in Belgium GPs are responsible for about 50% of repeat contraception prescriptions.

The strengths of the study were the representative size of the sample (1801 women), the use of a standardised counselling leaflet (prepared in cooperation with the European Society of Contraception and Reproductive Health), the guidance provided by a steering committee, and the extra survey conducted among the participating gynaecologists about the usefulness of the counselling method as advocated in this study.
